# Evaluation of Novel Targeted Therapies in Aggressive Biology Sarcoma Patients after progression from US FDA approved Therapies

**DOI:** 10.1038/srep35448

**Published:** 2016-10-17

**Authors:** Vivek Subbiah, Kenneth R. Hess, Muhammad Rizwan Khawaja, Michael J. Wagner, Chad Tang, Aung Naing, Siqing Fu, Filip Janku, Sarina Piha-Paul, Apostolia M. Tsimberidou, Cynthia E. Herzog, Joseph A. Ludwig, Shreyaskumar Patel, Vinod Ravi, Robert S. Benjamin, Funda Meric-Bernstam, David S. Hong

**Affiliations:** 1 Department of Investigational Cancer Therapeutics (A Phase I Program), Division of Cancer Medicine, The University of Texas MD Anderson Cancer Center, Houston 77030, Texas, USA; 2 Division of Pediatrics, The University of Texas MD Anderson Cancer Center, Houston 77030, Texas, USA; 3 Division of Biostatistics, The University of Texas MD Anderson Cancer Center, Houston 77030, Texas, USA; 4 Division of Radiation Oncology, The University of Texas MD Anderson Cancer Center, Houston 77030, Texas, USA; 5 Department of Sarcoma Medical Oncology, Division of Cancer Medicine, The University of Texas MD Anderson Cancer Center, Houston 77030, Texas.

## Abstract

Prognosis of patients with advanced sarcoma after progression from FDA approved therapies remains grim. In this study, clinical outcomes of 100 patients with advanced sarcoma who received treatment on novel targeted therapy trials were evaluated. Outcomes of interest included best response, clinical benefit rate, progression-free survival (PFS) and overall survival (OS). Median patient age was 48 years (range 14–80). Patients had received a median of 2 prior lines of systemic treatment. Phase I treatments were anti-VEGF–based (n = 45), mTOR inhibitor–based (n = 15), and anti-VEGF + mTOR inhibitor–based (n = 17) or involved other targets (n = 23). Best responses included partial response (n = 4) and stable disease (n = 57). Clinical benefit rate was 36% (95% confidence interval 27–46%). Median OS was 9.6 months (95% Confidence Interval 8.1–14.2); median PFS was 3.5 months (95% Confidence Interval 2.4–4.7). RMH prognostic score of 2 or 3 was associated with lower median OS (log-rank p-value < 0.0001) and PFS (log-rank p-value 0.0081). Receiving cytotoxic chemotherapy as part of phase I trial was also associated with shorter median OS (log-rank p-value 0.039). Patients with advanced sarcoma treated on phase I clinical trials had a clinical benefit rate of 36% and RMH score predicted survival.

Sarcoma accounts for only 0.9% of adult and 12% of childhood malignancies, with approximately 13,000 adult and 1,100 pediatric cases annually in the United States^1^,^2^. Sarcomas constitute a diverse class of molecularly distinct mesenchymal neoplasms of more than 50 subtypes^3^. Challenged by its rarity, heterogeneity, wide age range (straddling adult and pediatric oncology), complexity in chemotherapy and controversies, progress in systemic treatment for sarcoma has been relatively slow^4^. For the sake of simplicity, sarcomas can be grouped into two major categories either by location (e.g., bone vs. soft tissue sarcoma) or by presence or absence of genomic translocations that characterize one-third of sarcoma subtypes^5^,^6^.

The era of ‘-omics’ has helped reveal the complex biology of several sarcoma subtypes in terms of signaling pathways and molecular aberrations, thereby offering novel approaches to treatment by targeting aberrant pathways^7^. Successful targeting of activating mutations in the *KIT* receptor tyrosine kinase with imatinib mesylate for gastrointestinal stromal tumor (GIST) illustrates how this approach can potentially change outcomes even for notoriously chemotherapy-resistant sarcoma subtypes^8^,^9^. Sarcomas, especially those associated with a known translocation or those expressing a specific receptor, may be amenable to this approach with potentially exciting results. Although many preclinical studies with novel agents for sarcoma have shown promising results, the translation to bedside has been difficult given the rarity and diversity among sarcoma subtypes^10^,^11^,^12^. Conversely, clinical evaluation of investigational targeted agents for treatment of sarcoma may lead us to new pathways involved in sarcomagenesis^11^,^13^,^14^.

Phase I trials represent the most critical step in translation from bench to bedside^15^. Insulin-like growth factor type 1 receptor (IGF1R) inhibitors have demonstrated clear single-agent activity among patients with Ewing sarcoma in phase I trials^16^,^17^,^18^,^19^. Although more than 20 targeted agents - including monoclonal antibodies and small molecule inhibitors targeting IGF1R pathway with rationale for activity in sarcoma - were in various stages of development 5 years ago, the pharmaceutical industry lost enthusiasm for most of these agents because they were active in only rare subsets of sarcoma^20^,^21^. Predictive biomarkers are needed to identify the patients most likely to benefit from such targeted agents^22^. In the current study, we report the presenting characteristics and the outcomes of patients with sarcoma who were enrolled in phase I trials, primarily involving inhibitors of angiogenesis and mammalian target of rapamycin (mTOR), at The University of Texas MD Anderson Cancer Center (MDACC) and explore putative associations between patient characteristics and survival outcomes. In addition, we sought to validate the Royal Marsden Hospital (RMH) prognostic score among sarcoma patients enrolled in phase I clinical trials, as this score can help in patient prognostication^23^,^24^.

## Patients and Methods

### Data Collection and Pathology Review

We reviewed records of patients who were referred to the Phase I Clinical Trials Program at MDACC for refractory, relapsed, metastatic, or unresectable sarcoma. Patient characteristics and clinical outcomes were abstracted from transcribed notes in the electronic medical record system (ClinicStation, Houston, TX). Patient records were reviewed at the time of presentation to a phase I program. The type of investigational treatment regimens offered to patients varied throughout the study period given rapid protocol turnover. Outcomes of interest included objective response, stable or progressive disease, clinical benefit, and progression-free and overall survivals.

Patients who had a biopsy at another institution had their histopathologic findings verified by an MD Anderson pathologist. When biopsies were performed at MD Anderson, additional studies including cytogenetics, immunohistochemistry, fluorescent *in situ* hybridization, and/or polymerase chain reaction (PCR) were obtained as indicated. For some patients, mutational analysis was performed during the latter course of phase I trials (from 2008 onwards) if additional samples were available; mutations of interest included those in *KRAS*, *BRAF*, *C-KIT*, *EGFR*, and *P13KCA* genes.

All patients provided written informed consent before enrollment in phase I trials and all trials were approved and were carried out in accordance with the guidelines by the Institutional Review Board of MD Anderson Cancer Center, which also approved and granted waivers of informed consent for this retrospective study.

### Patient Eligibility

Eligible patients with metastatic or unresectable sarcoma, for whom approved curative therapies had failed, were included in the study. All patients had evidence of measurable disease according to the Response Evaluation Criteria in Solid Tumors (RECIST) criteria, Eastern Cooperative Oncology Group (ECOG) performance status of 0 to 2, and a life expectancy of at least 3 months. Premenopausal women were required to have negative results on a pregnancy test and patients with child-bearing potential were required to use contraception. A washout period of 4 weeks was required preceding initiation of treatment on phase I trial.

### Treatment and Follow-up

Once enrolled on a phase I trial, patients were evaluated at 3- to 4-week intervals in accordance with each protocol’s cycle length. At each visit, history was updated and physical examination was performed along with a comprehensive metabolic and hematologic panel. Patients were assessed for onset of new symptoms and compliance with treatment. Computed tomography (CT) or positron emission - computed tomography (PET-CT) scans were obtained every 2 cycles of therapy, and responses were determined based on RECIST criteria based on the protocol.

### Statistical Methods and End Points

Descriptive statistics were used to summarize the patients’ characteristics. Clinical benefit was defined as objective response or stable disease lasting 6 months or longer. The Kaplan-Meier method was used to estimate overall survival (OS) and progression-free survival (PFS) distributions and the log rank test was used to compare OS and PFS distributions between groups. Cox proportional hazards regression analysis was used to estimate the hazard ratio with 95% confidence intervals. OS was measured from date of presentation to the Phase I Clinical Trials Program until death from any cause or last follow-up. PFS was measured from date of study enrollment until disease progression or death (whichever came first) or last follow-up. A *P*-value of 0.05 or less was considered the criterion for statistical significance. Statistical analyses were performed using SAS 9.1 (SAS Institute, Cary, NC) and S-Plus software (version 7.0; Insightful Corp., Seattle, WA).

## Results

### Patient Characteristics

Table 1 describes the characteristics of the patients. One hundred patients (46 male, 54 female) with soft tissue sarcoma (n = 79) or bone sarcoma (n = 21) were included in the study. Median age of patients was 48 years (range 14–80). Patients received a median of 2 (range 0–10, interquartile range 1–4) prior lines of systemic therapies. Fifty-nine patients had received prior cytotoxic anthracyclines, 26 received topoisomerase inhibitors, 8 received platinum, 55 received antimetabolites, 63 received anti-mitotics, 63 received alkylating agents, and 15 patients had received other chemotherapy. Twenty-four patients had received agents targeting angiogenesis, 2 targeting HER2/neu, 1 EGFR, 1 CMET, 13 the PIK3CA/mTOR/AKT pathway, 5 C-KIT, and 15 patients had received other targeted agents. Five patients had received prior immunotherapy.

### Sarcoma Subtypes

A wide variety of sarcoma subtypes were treated (Table 2). Chondrosarcoma was the most common subtype (n =  9) among bone sarcomas, followed by Ewing sarcoma (n = 8). Spindle cell sarcoma was the most common soft tissue sarcoma (n = 12), followed by leiomyosarcoma (n = 10). Other soft tissue sarcomas included one patient each with adrenal sarcomatoid carcinoma, alveolar rhabdomyosarcoma, chondroid syringoma, chordoma, endometrial stromal sarcoma, epithelioid sarcoma, lymphangiomyomatosis, myoepithelial carcinoma, and unclassified primitive small cell malignancy.

### Molecular Aberrations

Table 3 describes the chromosomal rearrangements observed by sarcoma subtype. Other molecular abnormalities included *MET* mutation (7 of 50 patients), *HER2* amplification by fluorescence *in situ* hybridization (1 of 26 patients), *K-RAS* G12A mutation (1 of 67 patients), *N-RAS* Q61K mutation (1 of 45 patients), *EGFR* G719D mutation (1 of 55 patients), *P53* mutation (7 of 36 patients), and *C-KIT* mutation (1 of 56 patients). No MET amplification (62 patients), PIK3CA mutation (81 patients), or BRAF mutation (71 patients) was detected.

### Phase I Therapies and Outcomes

Table 4 describes the phase I treatments received by the patients included in this study. Forty-five patients received anti–vascular endothelial growth factor (VEGF)-based therapy, 15 received mTOR inhibitor–based therapy, 17 received anti-VEGF plus mTOR inhibitor–based therapy, and 23 patients received therapies based on targeted agents. Chemotherapy was a component of the clinical trial treatment in 30 patients. Best responses to phase I treatment included partial response in 4 patients, stable disease in 57, and progression in 39 patients. Thirty-two patients had stable disease for 6 months or more. Clinical benefit rate was 36% (95% CI: 27–46%). The four patients with partial responses included one patient with alveolar soft part sarcoma treated with an anti-VEGF agent, one with malignant fibrous histiocytoma treated with a combination of inhibitors of VEGF and histone deacetylase (HDAC), one with chondrosarcoma treated with a TRAIL (TNF-related apoptosis-inducing ligand) agent, and one patient with lymphangiomyomatosis treated with a combination of VEGF and mTOR inhibitors. None of these 4 patients harbored any targetable mutation.

**Figure 1:**
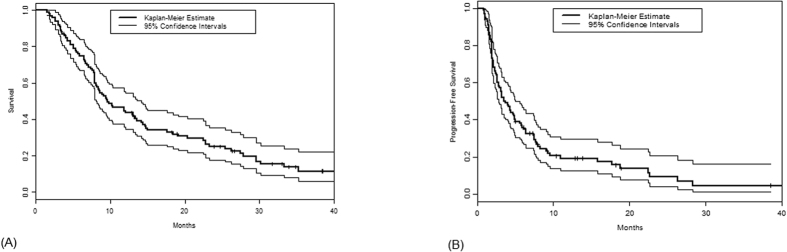
Overall survival (**A**) and Progression-free survival (PFS) among sarcoma patients enrolled on phase I trials.

**Figure 2:**
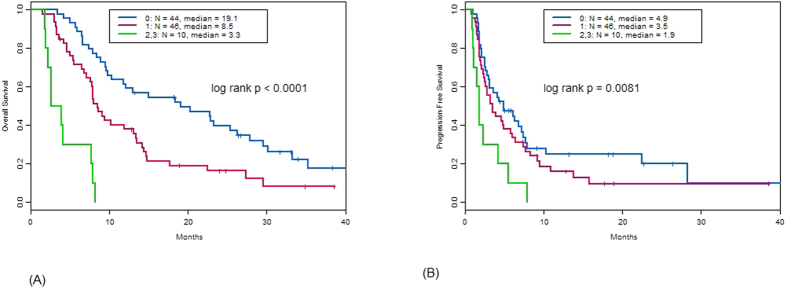
Overall survival (**A**) and Progression-free survival (PFS) among sarcoma patients enrolled on phase I trials with respect to Royal Marsden Hospital prognostic score.

Among the 100 patients in this study, 82 died after a median follow-up of 35 months. The median OS was 9.6 months (95% confidence interval: 8.1–14.2) (Fig. 1). Survival was 75% (95% CI: 67–84%) at 6 months, 44% (95% CI: 36–56%) at 1 year, 25% (95% CI: 18–36%) at 2 years, and 12% (95% CI: 6–22%) at 3 years. Eighty-three patients had disease progression on phase I treatment and 3 others died on study, for a total of 86 PFS events. Four patients came off trial by withdrawing consent, 3 due to toxicity, and 1 each for planned surgery and radiation therapy; 5 patients were continuing on phase I treatment. The median PFS was 3.5 months (95% confidence interval: 2.4–4.7) (Fig. 2). The PFS was 57% (95% CI: 48–68%) at 3 months, 36% (95% CI: 28–47%) at 6 months, 24% (95% CI: 17–35%) at 9 months, 19% (95% CI: 13–29%) at 1 year, 9% (95% CI: 4–20%) at 2 years, and 5% (95% CI: 1–16%) at 3 years. Table 5 presents the analyses of PFS and OS in various subsets of patients. Overall survival were better among patients with fewer than 3 metastatic sites, normal lactate dehydrogenase (LDH) values, and/or normal albumin values. The RMH prognostic score, a standardized system that includes these 3 parameters, was associated with PFS (log-rank p-value 0.0081) and OS (log-rank p-value < 0.0001). Patients who received cytotoxic chemotherapy as a component of their phase I trial treatment had shorter OS than those who did not receive cytotoxic chemotherapy (log-rank p-value 0.039).

## Discussion

This study summarizes the clinical outcomes of patients with refractory sarcomas in phase I clinical studies after progression from standard US FDA approved therapies. As we continue to decipher the unique molecular characteristics of these subtypes, phase I trials provide an opportunity to target these molecular characteristics to potentially induce responses. Improvement in treatment of GIST represents an excellent example. Before 2000, cases of GIST were often treated using regimens similar to those for leiomyosarcoma and were highly resistant to cytotoxic chemotherapy^25^,^26^. Identification of activation of the KIT oncogene in patients with GIST led to interest in its role in pathogenesis and the possibility of targeting this oncogenic driver^27^,^28^. A phase I study of imatinib, a known inhibitor of the KIT receptor tyrosine kinase, demonstrated an objective response rate of approximately 70%^29^.

However, the success in treatment of GIST has not been reproducible in all subtypes of sarcoma. For instance, despite a better understanding of the molecular pathogenesis of Ewing sarcoma, systemic chemotherapy remains the mainstay of treatment. The EWS-FLI1 fusion protein, the hallmark of Ewing sarcoma, positively regulates expression of IGF1R, which is necessary for fibroblast transformation^30^,^31^,^32^. However, early phase clinical trials of IGF1R inhibitors demonstrated response rates of 10–15%^33^,^34^,^35^ and highlighted the need for markers to identify patients most likely to respond.

Review of literature shows that a similar analysis among sarcoma patients enrolled in phase 1 clinical trials was conducted at the Royal Marsden hospital^36^. The median progression-free survival was 2.1 months (95% CI, 1.7–2.5), and median overall survival was 7.6 months (95% CI, 4.8–10.4)^36^. Another study from Europe reported the pooled analysis of 178 patients from the European database and reported similar results^37^. The similarities and differences are outlined in Table 6. Our current study closely mirrored the European database study.

Our study involved a diverse range of sarcoma subtypes in patients enrolled in a wide variety of phase I trials at MD Anderson Cancer Center. Neither demographic features, translocation status, nor mutations in TP53 or MET genes predicted outcome. The choice of therapy whether one used VEGF inhibitor, mTOR inhibitor, or other targeted agents similarly failed to significantly affect outcome. Paradoxically, the use of cytotoxic chemotherapy as part of phase I treatment was associated with shorter survival. This unexpected outcome may be due to the heavily pretreated nature of our phase I population. As most of these patients had progressed on chemotherapy before referral to phase I program, reduced survival in chemotherapy-treated group may reflect acquired drug resistance by patients’ tumors that predestined phase I agents to fail. Conversely, the use of biologically targeted therapies may have offered patients a novel mechanism of action not previously encountered as part of their standard-of-care regimens. It is noteworthy that chemotherapy was used in combination with inhibitor(s) of VEGF, mTOR, and/or other targets as part of phase I trials. We closely analyzed patients with an objective response to identify any distinguishing feature. Although 3 of the 4 patients with objective response received VEGF inhibitor, there was no difference in outcome of patients treated with VEGF inhibitor in the overall sample.

The initial report of the RMH prognostic score included only a few patients with sarcoma^23^. Although we previously validated use of the RMH score among patients with other cancers^24^,^38^, it was unclear whether the RMH score would be a valid tool for sarcoma patients. We sought to validate the RMH prognostic score among patients with sarcoma and found it to be a significant predictor of outcome.

This study has a few limitations that should be considered while interpreting its findings. First, the retrospective study design may have led to selection bias. Second, patients included were treated on phase I trials at a single institution, making the study susceptible to referral bias. Third, treatment involved inhibitors of VEGF and/or mTOR in more than three-quarters of the patients and no patients were enrolled on immunotherapy trials. While phase 1 trials are designed to evaluate toxicity not efficacy, the results shown here in should be viewed as preliminary. This is a retrospective study of sarcoma patients in several “all comer Phase 1” trials so we may not be able to derive any conclusions. Although efficacy activity is not the end point or primary objective in any Phase 1 trial, the primary motivation for patient’s participation and the treating physicians enrollment in phase I trials is the anticipation or optimism of some response/efficacy or therapeutic benefit^37^,^39^,^40^. Fourth, the survival for different sarcoma subtypes may be different. However, given the very low numbers we have to pool them together for this analysis for some meaningful analysis. In spite of aforementioned limitations, this study is valuable as it rigorously evaluated the largest experience in the USA with investigational cancer therapeutics among sarcoma patients as the rest of the studies have been mainly from Europe.

In conclusion, this retrospective analysis of 100 sarcoma patients treated on phase I trials predominantly involving inhibitors of VEGF and/or mTOR demonstrated a clinical benefit rate of 36%. Higher RMH score and treatment with cytotoxic chemotherapy as part of phase I trial were associated with shorter OS.

## Additional Information

**How to cite this article**: Subbiah, V. *et al.* Evaluation of Novel Targeted Therapies in Aggressive Biology Sarcoma Patients after progression from US FDA approved Therapies. *Sci. Rep.* **6**, 35448; doi: 10.1038/srep35448 (2016).

## Figures and Tables

**Table 1:** Patient characteristics of sarcoma patients enrolled on phase I trials.

Characteristic	Total (N = 100)	Soft Tissue Sarcoma (N = 79)	Bone Sarcoma (N = 21)
Sex			
Male	46	35	11
Female	54	44	10
Age (years)			
Median (range)	48 (14–80)	49 (16–80)	29 (14–70)
Ethnicity			
White	76	58	18
Black	13	12	1
Hispanic	8	6	2
Asian	3	3	0
No. of metastatic sites			
<3	62	48	14
≥3	38	31	7
LDH			
≤ULN	79	61	18
>ULN	21	18	3
Albumin			
≥3.5 g/dL	92	71	21
<3.5 g/dL	8	8	0
No. of prior therapies			
<3	51	42	9
≥3	49	37	12

Abbreviations: LDH, lactate dehydrogenase; ULN, upper limit of normal.

**Table 2:** Sarcoma subtypes among patients enrolled on phase I trials.

Bone Sarcoma (N = 21)	
Chondrosarcoma	9
Ewing’s sarcoma	8
Osteosarcoma	4
Soft Tissue Sarcoma (N = 79)	
Spindle cell sarcoma	12
Leiomyosarcoma	10
Synovia sarcoma	7
Clear cell sarcoma	7
Alveolar soft part sarcoma (ASPS)	5
Liposarcoma	5
Desmoplastic small round cell tumor	5
Malignant fibrous histiocytoma	4
PEComa	3
Hemangiopericytoma/fibrous sarcoma	3
Gastrointestinal stromal tumor (GIST)	2
Angiosarcoma	2
Unclassified high-grade sarcoma	5
Other subtypes (1 each) included adrenal sarcomatoid carcinoma, alveolar rhabdomyosarcoma, chondroid syringoma, chordoma, endometrial stromal sarcoma, epithelioid sarcoma, lymphangiomyomatosis, myoepithelial carcinoma, and unclassified primitive small cell malignancy	9

**Table 3:** Chromosomal Rearrangements by Sarcoma Subtype.

Rearrangement	Sarcoma Subtype	No. of Patients
EWSR1:FLI1	Ewing sarcoma	8
EWSR1	Clear cell sarcoma	6
EWS:WT1	Desmoplastic small round cell tumor	3
SYT-SSX	Synovial sarcoma	4
ASPL-TFE3	Alveolar soft part sarcoma (ASPS)	2
KIAA-BRAF	Spindle cell sarcoma	1
12q15 amplification	Liposarcoma	1

**Table 4:** Phase I treatments received by sarcoma patients.

Phase I Treatment	No. of Patients	Bone Sarcoma	Soft Tissue Sarcoma
Anti-VEGF based			
VEGF only	10	0	10
VEGF + chemo	7	1	6
VEGF + immunomodulator	8	0	8
VEGF + targeted	8	2	6
VEGF + targeted+ chemo	6	1	5
VEGF + HDAC	6	2	4
mTOR-inhibitor based			
mTOR alone	2	0	2
mTOR + chemo	2	1	1
mTOR + immunomodulator	2	0	2
mTOR + targeted	5	2	3
mTOR + HDAC	1	1	0
mTOR + chemo+ proteosome	3	0	3
Anti-VEGF + mTOR-inhibitor based			
VEGF + mTOR	7	2	5
VEGF + mTOR + chemo	9	1	8
VEGF + mTOR + targeted	1	1	0
Target based			
Targeted	20	6	14
Targeted + chemo	3	1	2

Abbreviations: chemo, chemotherapy; HDAC, histone deacetylase, mTOR, mechanistic target of rapamycin; VEGF, vascular endothelial growth factor.

**Table 5:** Survival Outcomes in Various Subsets of Sarcoma Patients.

	N	Median PFS (Months)	HR (95% CI)	Median OS (Months)	HR (95% CI)
Sex					
Female	54	4.3	0.68 (0.44–1.04)	10.2	0.73 (0.47–1.13)
Male	46	3.1	Reference	8.6	Reference
Age					
<40 years		3.7	0.96 (0.62–1.49)	9.5	0.98 (0.63–1.53)
≥41 years		3.5	Reference	9.8	Reference
Sarcoma subtype					
Soft tissue	79	4.3	0.80 (0.47–1.35)	10.4	0.90 (0.53–1.52)
Bone	21	2.9	Reference	7.9	Reference
Translocation					
Present	25	3.6	1.0 (0.61–1.63)	8.4	1.21 (0.74–1.98)
Absent	75	3.5	Reference	9.8	Reference
MET mutation					
Present	7	5.0	0.99 (0.44–2.23)	14.2	1.15 (0.51–2.62)
Absent	43	3.2	Reference	9.1	Reference
P53 mutation					
Present	7	6.4	0.79 (0.32–1.96)	14.7	0.89 (0.36–2.18)
Absent	29	4.3	Reference	11.8	1.13 (0.46–2.78)
Phase I treatment					
Anti-VEGF	62	4.0	0.95 (0.61–1.47)	9.5	1.31 (0.83–2.08)
No anti-VEGF	38	3.2	Reference	9.8	Reference
mTOR inhibitor	32	4.9	0.86 (0.54–1.36)	10.2	0.69 (0.42–1.12)
No mTOR inhibitor	68	3.2	Reference	9.4	Reference
Target inhibitor	38	3.2	0.98 (0.63–1.52)	9.6	1.0 (0.65–1.58)
No target inhibitor	62	3.7	Reference	9.5	Reference
Chemotherapy	30	2.9	1.32 (0.83–2.10)	7.9	1.63 (1.02–2.60)
No chemotherapy	70	4.4	Reference	13.0	Reference
Prior lines of treatment					
2 or less	51	4.1	0.94 (0.62–1.45)	11.8	0.86 (0.56–1.33)
3 or more	49	3.2	Reference	8.6	Reference
No. of metastatic sites					
2 or less	62	3.1	0.91 (0.58–1.41)	12.1	0.58 (0.37–0.91)
3 or more	38	4.3	Reference	7.9	Reference
LDH					
Normal	79	4.4	0.55 (0.33–0.91)	12.1	0.46 (0.28–0.78)
High	21	2.3	Reference	7.9	Reference
Albumin					
Normal	92	4.0	0.28 (0.13–0.60)	11.8	0.20 (0.09–0.44)
Low	8	1.6	Reference	3.9	Reference
RMH prognostic score					
0	44	4.9	0.33 (0.16–0.67)	19.1	0.10 (0.05–0.23)
1	46	3.5	0.46 (0.23–0.93)	8.5	0.21 (0.10–0.44)
2–3	10	1.9	Reference	3.3	Reference

Abbreviations: LDH, lactate dehydrogenase; mTOR, mechanistic target of rapamycin; RMH, Royal Marsden Hospital; VEGF, vascular endothelial growth factor.

**Table 6:** Similarities and differences among the three major Phase 1 trial experiences with advanced sarcoma patients on Phase 1 trials.

	MDACC Phase 1 unit	Royal Marsden Hospital Phase 1 unit^36^	European Phase 1 database^37^
Number of patients	100	133	178
Median no. of systemic therapies	2 (0–10)	3 (0–6)	2 (0–9)
Age range	48 (14–80)	48.0 (12.5–81.9)	51 (19–79)
Male/Female ratio	46/54	71/62	96/82
Best Responses	4 PR	1 CR, 2PR.	6 PR
PFS	median PFS 3.5 months (95% CI: 2.4–4.7) 3 month and 6 month PFS, 57% and 36%	Median PFS 2.1 months (95% CI, 1.7–2.5)	3-month and 6-month PFS rates were 33.5% and 16.9%
Median OS	9.6 months (95% CI: 8.1–14.2)	7.6 months (95% CI, 4.8–10.4).	9.8 months (39.4 weeks) (95% CI 27.4–51.4)
Major tumor types	Spindle cell Sarcoma 12 (12%), Leiomyosarcoma 10 (10%)	Leiomyosarcoma 16 (12%), GIST 15 (11.3%), Liposarcoma 15 (11.3%)	Leiomyosarcoma 34 (27.2%), Liposarcoma 16 (12.8%)
Major drug types	anti-VEGF–based (n = 45), mTOR inhibitor–based (n = 15), and anti-VEGF + mTOR inhibitor–based (n = 17)	Antiangiogenic 43 (32.3%), IGF1-R/PI3K/mTOR/AKT pathway 30 (22.6%)	Cytotoxic 43 (19.8%), Targeted or cytotoxic+ targeted 174 (80.2%)
